# Pre-trauma Methylphenidate in rats reduces PTSD-like reactions one month later

**DOI:** 10.1038/tp.2016.277

**Published:** 2017-01-10

**Authors:** G Ritov, G Richter-Levin

**Affiliations:** 1 The Institute for the Study of Affective Neuroscience, University of Haifa, Haifa, Israel; 2 Sagol Department of Neurobiology, University of Haifa, Haifa, Israel; 3 Department of Neurobiology, Weizmann Institute of Science, Rehovot, Israel; 4 Psychology Department, University of Haifa, Haifa, Israel

## Abstract

In basic research, the etiology of fear-related pathologies, such as post-traumatic stress disorder (PTSD), is conceptualized using fear-conditioning protocols that pair environmental stimuli (that is, a conditioned stimulus—CS) with an aversive, unconditioned stimulus (US) to elicit an assessable conditioned fear response. Although pathophysiological models agree that regulatory dysfunctions in this associative process may instigate fear-related pathology, current opinions differ in regard to the nature of these dysfunctions. Primarily derived from studies in rodents, the prevailing perspective proposes that pathological fear-reactions develop from intensified and overly consolidated CS-US associations. Alternatively, models derived from studies in humans suggest that tempospatial inaccuracies in representations of associative fear might precipitate pathology by engendering failure to differentiate present experiences and past memories of threat. To test this concept in rodents, we administered rats with cognition enhancing doses of Methylphenidate before or after fear conditioning and measured long-term alterations in their conditioned fear behaviors and PTSD-like reactions. The administration of Methylphenidate before fear-memory formation indeed reduced anxious-like responses during fear-memory retrieval one month later. An individual profiling analysis revealed that Methylphenidate onset had opposing effects on the risk for PTSD-like classification. The modulation of initial learning and formation of associative fear normalized the risk for developing PTSD-like reaction. In contrast, when the effects of Methylphenidate were exerted only over later consolidation this risk increased markedly. When examined under current psychiatric and neuropharmacologic literature, these results reveal a possible strategy of using low-dose Methylphenidate for the prevention of PTSD in high risk populations.

## Introduction

Fear learning is an adaptive process that allows stimuli preceding an aversive outcome to acquire the capacity to signal danger and elicit defensive responses against potential future threats. Although essential for generating appropriate responses in altering environments, this memory process is highly sensitive to increasing levels of stress.^1^ Contextual stimuli associated with an extremely aversive experience can in fact become pathogenic by provoking dysregulated fear responses in later harmless situations.^2^ Indeed, irrepressible fear and anxious responses to reminders of stressful events are hallmark features of fear-related pathologies such as anxiety, phobias and post-traumatic stress disorder (PTSD).^3^ As in many cases of fear-related disorders, particularly PTSD, the dysregulated fear response is initiated by a distinct incident, neuropsychiatric research focus on mechanisms mediating the process of fear learning and memory.^4^ Thus, in laboratory studies, the etiology of fear-related disorders and its neural substrates is often conceptualized using fear-conditioning protocols that pair environmental stimuli (that is, a conditioned stimulus—CS) with an aversive, unconditioned stimulus (US) to elicit an assessable conditioned fear response (CR). Pathophysiological models identify the amygdala, hippocampus and medial prefrontal cortex (mPFC) as key constituents in the neural networks mediating this classical learning, and generally agree that regulatory dysfunctions in associative processing within this circuitry are at the core of pathological fear behavior occurring in fear-related disorders.^4, 5, 6, 7, 8^ However, current opinions differ in regard to the nature of the dysfunction and its specific effects on fear memory formation and modulation.

Centering on the amygdala as a site of CS–US convergence and initiation of fear response, the dominant view asserts that enhanced acquisition and consolidation of aversive associations precipitate the development of fear-related disorders. Primarily derived from fear conditioning studies in rodents, this viewpoint proposes that exaggerated amygdala responses to aversive inputs critically strengthen CS-US pairings,^1, 6, 7^ producing overly consolidated and potentially pathogenic representations of associative fear.^2, 4^ With time, this associative imbalance is suggested to impair CR regulation and mPFC capacity to inhibit excessive fear-responses to the CS features.^2, 4, 5, 6, 7^

Deviating from these conventional conceptions, recent findings of neuroimaging studies in humans provide evidence that aversive inputs can modulate corticolimbic processing of the CS features during conditioning,^8^ and in fear-related disorders, induce critical changes in core representations of the CS space.^9^ Such studies give rise to an alternative perspective that emphasizes the role of the memory’s accurateness in the pathogenesis of fear-related disorders.^10^ According to this perspective, aversive US inputs modify basic processes of associative encoding during emotional learning, and in extreme situations, impair the precision of tempospatial representations via hippocampal plasticity.^11^ Considering the role of the hippocampus in pattern separation,^12^ these imprecisions are suggested to engender failure to differentiate present experiences and past memories of threat and thus provoke pathology in liable individuals.^11^ Yet, this alternative perspective lacks the resolution and causativeness provided by animal research. To bridge this gap, we have recently developed a reverse-translation model that enables to identify dysregulated patterns of fear-behaviors and PTSD-like reactions in rodents.^13^ In this model, rats are initially habituated to the context of the water associated zero maze (WAZM) for 4 consecutive days and form a safe representation of its environment. Following the 4th day habituation, rats are exposed to an extremely aversive underwater trauma (US) in the aquatic center of the WAZM and form a new fear-associated representation for its context (CS). One month later, rats are presented with this CS to measure long-term alterations in different fear responses and an individual profiling approach is then implemented to classify PTSD-like individuals. Combining this novel protocol with microanatomical analyses of cellular activation, we uncovered an extremely anxious sub-group, within the stress-exposed population, demonstrating dysregulated patterns of excitation and co-activity in the mPFC–amygdala–hippocampus circuit.^13^ Importantly, these patterns exposed a distinct imbalance within the hippocampus of the anxious individuals, indicating an intrinsic desynchronization during fear memory retrieval between its dorsal division (DH), which subserves tempospatial memory processing,^12, 14^ and its ventral division (VH) which mediates fear memory and anxiety.^12, 14^

As our neuroanatomical data, correlating fear-related pathology with tempo-emotional impairments in hippocampal representations of aversive associations,^13^ seemed to concur with the alternative perspective above, we set out to evaluate its predictions by combining our behavioral model with pharmacological manipulations of fear memory formation. To this end, we took advantage of Methylphenidate (MPH), a selective dopamine (DA) and noradrenaline (NA) reuptake inhibitor that is commonly prescribed for the treatment of attention deficit hyperactivity disorder (ADHD). Although MPH increases the expression of extracellular catecholamines, at clinically relevant low doses it can improve cortical cognitive function while exerting only modest effects in neural circuits associated with arousal and pleasure.^15^ In nonclinical samples, low-dose MPH was shown to improve working memory performance and consolidation of long-term representations in both healthy individuals^16, 17, 18^ and laboratory rats.^19, 20^ These low-dose cognition-enhancing effects are suggested to result from strengthening of signal processing within the PFC,^15^ and synaptic plasticity within the hippocampus,^21^ via preferential activation of different NA (α/β) and DA (D1/D5) receptors.

To examine whether the accuracy of representations of an aversive association contributes to the development of fear-related pathology, in this study we treated rats with procognitive doses of MPH (0.5 mg/kg P.O.) at different stages of fear-memory processing. As in rats extracellular catecholamines reach peak concentration within 40 min of oral MPH onset,^15, 19^ we administered MPH 1 h before, or after, the safe context of the WAZM was paired with the underwater trauma experience. Given its memory enhancing effects, we hypothesized that MPH administration before the acquisition of this aversive association may reduce inaccuracies in its tempospatial–emotional representation, and by this, reduce pathological responses to its reminders in the long-term.

## Materials and methods

### Subjects and experimental timeline

**Figure 1:**
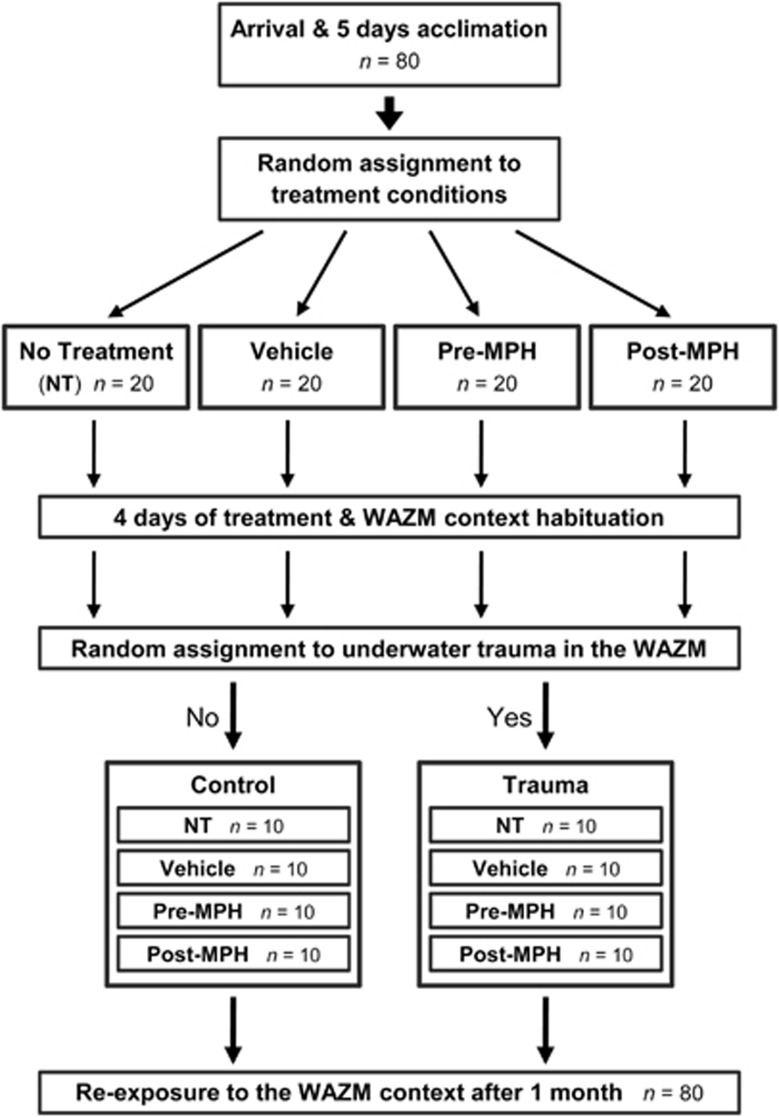
Experimental design. Following arrival and acclimation all rats were randomly assigned to one of four conditions: No Treatment (NT), Vehicle, Pre-MPH and Post-MPH. In the Pre-MPH and Vehicle conditions the treatment solution was orally administered 1 h before the WAZM tests, and in the Post-MPH condition 1 h after. Treatments were administered only during the initial 4 days of the experiment. Immediately after the 4th day test, 50% of the rats in each treatment condition were randomly assigned to the underwater trauma stress of 45 s. restrain under water in the WAZM center. One month later all rats were re-exposed to the WAZM test as a contextual reminder. MPH, methylphenidate; WAZM, water-associated zero maze.

All experimental procedures were performed adhered to the NIH Guide for the care and use of laboratory animals and were approved by the University of Haifa ethical committee. This study used 80 adult male Sprague Dawley (SD) rats (8–9 weeks old) weighing 250–275 g (Harlan, Jerusalem, Israel) at arrival. Animals were housed two per cage in a temperature-controlled (23±1 °C) animal facility on a 12:12 h light–dark cycle (lights on 0700–1900 hours). They had ad libitum access to standard rodent chow pellets and water. Throughout the study, identical procedures were repetitively performed for five times on consecutive batches (*n*=16 each). As illustrated in Figure 1, following arrival and acclimation all rats were randomly assigned to one of four conditions (*n*=20 in each condition): no treatment (NT), Vehicle, Pre-MPH and Post-MPH. Rats were then habituated to the WAZM test and the oral administration of MPH and vehicle for 4 consecutive days. In the Pre-MPH condition, MPH was administered 1 h prior to the WAZM tests. In the Post-MPH condition MPH was administered 1 h after the WAZM tests. In the Vehicle condition, vehicle solution was administered 1 h prior to the WAZM tests. Habituation procedures were performed between 1000 and 1700 hours. Immediately after the 4th day test, 50% of the rats in each condition were randomly assigned to the underwater trauma (UWT). In this trauma condition rats (*n*=40) were exposed to the WAZM test and immediately after to the UWT stress of 45 s restrain underwater in the WAZM center. In the control condition rats (*n*=40) were only exposed to WAZM test. The UWT exposures were consistently conducted between 1100 and 1400 hours throughout the experiments. One month later all rats were re-exposed to the WAZM testing as a contextual reminder by a blind investigator. On the basis of their behavior rats were classified as ‘PTSD-like’ individuals as described below.

### Pharmacological treatment

MPH and vehicle were orally administered during the initial 4 days of the experiment. For the MPH treatment (0.5 mg/kg), 10 mg Ritalin tablet (Novartis Farmacéutica, Barcelona, Spain) were grounded and fluidized in 10% sucrose in tap water solution to a 1 mg/ml concentration. For the vehicle, only the 10% sucrose in tap water solution was used. The solution (~75 μl) was then administered with a micropipette by placing the filled tip in or near the animal’s mouth and slowly discharging the suspension to allow the animal to drink (following Wheeler *et al.*^22^).

### Behavioral protocols

#### Water-associated zero maze

**Figure 2:**
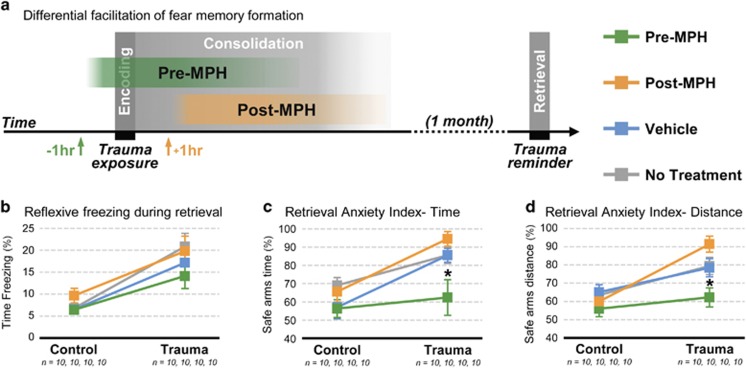
To test whether excessive fear-related responses develop from inaccurate representations of aversive associations, MPH was administrated to facilitate memory processing during associative fear conditioning and behavioral responses were assessed during fear memory retrieval one month later. (**a**) Logical concept design. (**b**) The administration of MPH before the encoding and consolidation of the aversive association did not increase reflexive fear responses during memory retrieval; nor did it block the basic learning of fear- all trauma groups significantly differed from the control groups. (**c**, **d**) Significantly differing from the rest of the trauma groups, the Pre-MPH trauma-exposed rats exhibited a similar to the control groups’ choice of risk behaviors during memory retrieval, as measured by the anxiety indices for (**c**) the ratio of time spend in the safe arms, and (**d**) the ratio of distance traveled in the safe arms of the WAZM. *n*, number of animals analyzed; *P<0.05 (Bonferroni *post hoc*). Error bars represent mean±s.e.m. MPH, Methylphenidate; WAZM, water associated zero maze.

The WAZM is a transformation of the elevated zero maze to an integrated wet and dry context (for further details of this apparatus see Ritov *et al.*^23^). This apparatus enables the formation of an association between the maze and an underwater trauma, and by that, the assessment of complex behaviors during re-exposure to the context that immediately precedes a traumatic experience. For the tests, rats were first habituated to the room for 2 min and then were placed into one of the open arms facing a closed part of the apparatus. Rats were allowed to explore the arena for a 5 min session. During this time rat’s behavior was tracked, recorded and analyzed by the Etho-Vision system (Noldus Information Technology, Wageningen, the Netherlands). Behavioral measures included the time spent in the open arms, distance traveled in the open arms, distance traveled in the closed arms and total freezing. Standard anxiety indices (Figures 2c and d) were measured as percentage of closed arms time (the ratio of time spent in the closed arms to open arms time × 100) and percentage of closed arms distance (the ratio of distance traveled in the closed arms to distance traveled in the open arms × 100).

### Underwater trauma stress

The UWT stress was carried out immediately after the 4th habituation. Trauma rats were lifted from the dry arms, placed in the aquatic center of the WAZM (containing water at 22±2 °C, 50 cm deep) and submerged under water for 45 s, using a special metal net (20 × 10 × 15 cm). After the procedure the animals were dried briefly and returned to their housing cages. This procedure is known to acutely and enduringly increase anxiety-like behavior^13, 23, 24, 25, 26, 27, 28^ without any tissue damage.^29^ The procedure did not cause any loss of life or changes in body weight compared with control.

### Behavioral profiling

This individual profiling analysis based on the within group variablility of animals behavior in order to identify behavioral reactions of dysregulated fear as was shown before.^13^ For this, anxious-like responses were categorized according to the distribution of the behvior of the no-treatment, no-trauma, group (Control NT) during the WAZM re-exposure. Classification criteria were based on the lower 20th percentiles of Control NT's distribution for time spent in the open arms, distance traveled in the open arms and distance traveled in the closed arms. The freezing criterion based on the upper 80th percentile of control NT's distribution. All rats were individually discerned for each criterion. For the final classification, every rat that demonstrated a behavioral profile that fell within a minimum of 3 out of 4 criteria was classified as a PTSD-like rat.

### Statistical analysis

Data were analyzed using the IBM SPSS Statistics Software (IBM, Armonk, NY, USA). Shapiro–Wilk test of normality and Levene’s test for homogeneity of variance were utilized for inclusion in parametric tests. No animals were excluded from the behavioral analyses. A 2 × 4 × 3 multivariate analysis of variance (MANOVA) was used to examine the effects of UWT (Trauma/Control) and treatment (NT, Vehicle, Pre-MPH and Post-MPH) on the standard anxiety indices of time, distance and percentage of freezing. Significant interactions were followed by Bonferroni *post hoc* comparisons.

## Results

To test whether excessive fear-related responses develop from inaccurate representations of aversive associations, MPH was administrated to facilitate memory processing during the pairing of a previously safe context with an underwater trauma (Figure 2a). No differences were found in the behaviors of control and trauma rats in the different conditions before this pairing (Supplementary Table S1). One month later behavioral responses were assessed during fear memory retrieval. An initial 2 × 4 × 3 MANOVA was used to evaluate the long-term effects of the trauma exposure, the pharmacological treatment and their interaction on the reflexive fear behavior of freezing and the anxiety indices for time and distance. This analysis found significant multivariate main effects for the trauma exposure (Wilks’ *λ*=0.543, F_(3,70)_=19.62, *P*<0.001), the pharmacological treatment (Wilks’ *λ*=0.751, F_(9,170.51)_=2.37, *P*=0.015) and their interaction (Wilks’ *λ*=0.786, F_(9,170.51)_=1.98, *P*=0.045). As shown in Figures 2b–d, the administration of MPH before the initial encoding of the aversive conditioning did not increase CR expression to the presentation of the CS 1 month later. Nor did it block the basic learning of fear. Pre-MPH trauma-exposed rats did not differ from the rest of the trauma-exposed rats in reflexive freezing; and all trauma groups exhibited a significant increase in reflexive freezing in comparison with the control groups (Figure 2b). However, the administration of MPH before the early formation of representation of the aversive association, but not during later stages of its consolidation, specifically affected the Pre-MPH trauma-rat’s choices of risk and anxious-like behaviors during fear memory retrieval. Significantly differing from the rest of the trauma groups (*P*<0.05, Bonferroni *post hoc*), the Pre-MPH trauma-exposed rats exhibited a similar to the control groups choice of risk behaviors as measured by the anxiety indices for the ratio of time spend in the safe arms (Figure 2c) and the ratio of distance traveled in the safe arms (Figure 2d).

**Figure 3:**
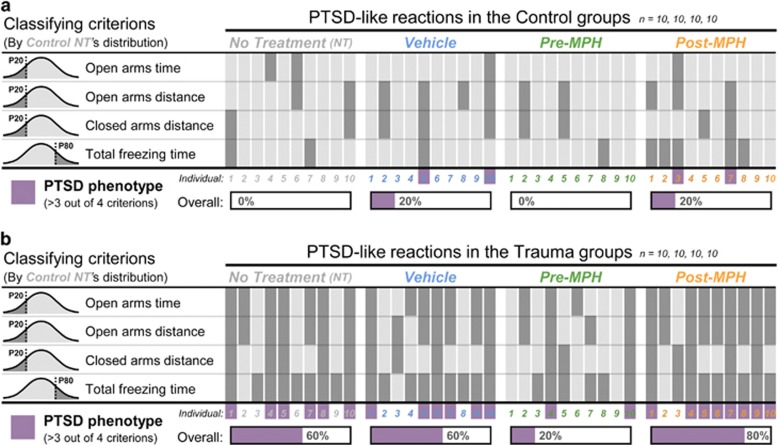
Individual profiling analysis for post-traumatic stress disorder (PTSD)-like reactions. To evaluate the prevalence of rats exhibiting PTSD-like reactions in each of the experimental conditions, profiling criteria were defined according to the lower or upper 20th percentiles of the distribution of the behvior of the no-treatment, no-trauma, group (Control NT). All rats were individually discerned for each criterion and every rat that demonstrated a behavioral profile that fell within a minimum of 3 out of 4 criterions was classified as a PTSD-like rat. (**a**) The prevalence of PTSD-like reactions in the control groups. (**b**) The trauma exposure alone increased the prevalence of PTSD-like reactions significantly, yet the MPH treatment induced a differential effect. Pre-MPH reduced the risk for PTSD-like classification whereas Post-MPH increased this risk. *n*, number of animals analyzed; *P*, control NT’s percentile; MPH, Methylphenidate.

Next, we used an individual profiling approach to evaluate the prevalence of rats exhibiting PTSD-like reactions in each of the experimental conditions. The profiling criterions were defined according to the distribution of the behvior of the no-treatment, no-trauma, group (Control NT). Classification criteria based on the lower 20th percentiles of Control NT's distribution for time spent in the open arms (<59.4 s), distance traveled in the open arms (<295 cm) and distance traveled in the closed arms (<507 cm). The freezing criterion based on the upper 80th percentile of Control NT's distribution (>30.8 s). All rats were individually discerned for each criterion and every rat that demonstrated a behavioral profile that fell within a minimum of 3 out of 4 criterions was classified as a PTSD-like rat (Figure 3). In line with our recent findings,^13^ the trauma exposure alone increased the prevalence of PTSD-like reactions significantly (*χ*^2^_(7)_=28.9, *P*<0.001), with 60% of trauma-exposed rats in the no-treatment and Vehicle conditions qualifying for PTSD-like classification. The pharmacological treatment however induced a differential effect (Figure 3b). The administration of MPH before the encoding of the aversive association reduced the risk for PTSD-like reactions markedly, with only 20% of trauma-exposed rats in the Pre-MPH condition qualifying for PTSD-like classification. In contrast, MPH administration only during later stages of consolidation increased this risk, with 80% of trauma-exposed rats in the Post-MPH condition qualifying for PTSD-like classification.

## Discussion

To test if inaccuracies in the representations of aversive associations might contribute to the development of fear-related pathology, we combined a reverse-translation model of PTSD in rats with pharmacological manipulation of fear memory formation. We hypothesized that administration of cognition-enhancing doses^15^ of MPH before an aversive experience may acuminate its associative representations and consequently reduce pathological responses to its reminders. Supporting this hypothesis, our data demonstrated that administration of MPH before the encoding and consolidation of a traumatic experience actually reduces pathological fear responses to its associative reminders in the long term. When combined with the abnormal hippocampal patterns we have previously identified in highly anxious rats,^13^ our results emboldens the theoretical perspective proposing that incoherent representations of sensory modalities and emotional valence can promote less regulated reactions to trauma related cues^30^ and, as a result, fear-related pathology.^9, 10, 11^ Moreover, in line with this pathophysiological concept, our individual profiling analysis revealed that the Post-MPH had an opposite effect on the risk for PTSD-like classification. Thus, when the initial formation of associative fear representations was modulated by Pre-MPH, the risk for developing PTSD-like reaction was normalized. Yet strikingly, when the initial learning was not manipulated, and the effects of MPH were exerted only over the consolidation of the aversive representations, the risk for developing PTSD-like reaction increased markedly.

When considered under the common views in the field of behavioral neuroscience, our results reveal a possible inconsistency. We found that facilitation of memory formation during a fear conditioning protocol did not increase the expression of conditioned fear responses in the long term (Figure 2). Nonetheless, due to several methodological reasons, this intriguing paradox does not necessarily contradicts, but rather challenges research in the field. First, most animal models of fear-related disorders do not implement life-threatening stressors, such as the underwater trauma used here. Likewise, most rodent studies measure fear memory by assessing reflexive behaviors such as freezing and startle amplitude,^2, 4, 5, 6, 7^ yet here we used the WAZM, an apparatus that enables multiple behavioral measures during fear memory retrieval.^23^ Thus, had we solely relied on freezing assessment no substantial effects for Pre-MPH could be concluded, as all trauma-exposed rats demonstrated a significant increase in freezing in comparison with controls. Moreover, in the absence of this main effect on freezing, the results of the anxiety indices would be limited by the possible risk for Pre-MPH state dependent effects. Yet, the WAZM enabled us to assess reflexive freezing to ascertain that fear learning indeed occur, and as an indication of increased arousal, and in parallel, to assess additional behaviors that may be more relevant for indicating re-experiencing and avoidance-like symptoms.^23^ Finally, although providing priceless mechanistic insights into the circuitry underlying fear learning and memory, the majority of studies in rodents contrast exposed and non-exposed populations while individual variations within the exposed population draw less attention.^13^ Translationally wise, this is at odds with clinical data showing that only a subset of trauma-exposed individuals develops PTSD in the long term.^3, 13^ Our results thus emphasize the importance of accounting for inter-individual differences to better understand the psychophysiological processes transforming a ‘normal’ fear behavior to a pathologically dysregulated fear response.

In regard to potential therapeutic mechanisms of MPH in fear-related disorders, it was previously shown that post-exposure chronic administration of high-dose MPH (2.7 mg/kg intraperitoneally) can improve PTSD-like symptoms in rats long after stress exposure.^31^ However, to the best of our knowledge, this study provides first evidence that pre-trauma low-dose MPH may possibly prevent post-traumatic fear-related pathology. Furthermore, to avoid any reinforcing or anxiolytic effects, as seen with higher doses of MPH, here we used very low doses (that is, 0.5 mg/kg P.O. in rats) to exert qualitatively different, memory-enhancing effects with strong translational relevance.^15^ Regrettably, due to its psychopharmacological nature, our data cannot provide direct indications of the neurocircuits that could underlie the pre-trauma MPH amelioration of psychopathological post-traumatic symptoms. Nonetheless, the neurofunctional effects of low-dose MPH strongly associate with the neural correlates of fear-related disorders, particularly the ones involved in PTSD. When combined, independent observations from both humans and rodents can thus offer strong logical interpretations for possible micro- and macro-level mechanisms involved in the preventive effects of pre-trauma MPH.

In rodent research, the effects of MPH have been mostly studied in fronto-striatal circuits. Within the PFC, low doses of MPH are suggested to bias neuronal activation to task-relevant information, while simultaneously reducing responding to irrelevant stimuli, via specific activation of catecholamine signaling at α_2_ adrenergic and D1 dopaminergic receptors (see Spencer *et al.*^15^ for a comprehensive review). MPH is also suggested to affect glutamatergic signaling in the PFC via specific alterations in *N*-methyl-d-aspartate receptor (NMDAR) function.^32^ In the context of stress, MPH is suggested to restore stress-induced changes in PFC dendritic spine densities and resultant cognitive impairments.^33^ Thus, although stress exposure disrupts PFC dendritic growth^33^ and NMDAR-dependent information processing,^34^ post-exposure MPH was shown to reduce stress-induced impairments in PFC NMDAR^32^ function and restore cognitive performance.^32, 33^

In the hippocampus, stress-induced impairments in glutamatergic signaling have been suggested to contribute to the development of stress-related pathology.^35^ Yet, differing from the PFC pathways, within the hippocampus low-dose MPH was recently shown to strengthen signal transmission (that is, a sustained long-term potentiation (LTP)) via specific activation of β-adrenergic and D1/D5 dopaminergic receptors; and promotion of mobilization and insertion of AMPA receptors in synapses involved in recent patterns of activity.^21^ Given the differential effects of emotional stress on synaptic plasticity in the DH and VH,^14^ this specific involvement of β-adrenergic receptors in hippocampal LTP is extremely relevant to possible mechanisms involved in the protective effects of pre-trauma MPH. Although under normal conditions the ability to evoke LTP is lower in the VH compared with the DH, acute stress exposure reverse this completely, causing facilitation of LTP in the VH and its suppression in the DH.^36^ Over-regulating functional VH-amygdala routes during fear memory formation,^14, 36^ this dynamic plasticity is suggested to consequently produce the abnormal VH-amygdala synchronization involved in dysregulated fear responses during the retrieval of representations of aversive associations.^13, 23, 27^ Strikingly, the preferential target of MPH in the hippocampus, β-adrenergic receptors,^21^ are also predominantly involved in this stress-induced routing of synaptic plasticity,^37, 38^ and specific long-term underwater trauma-induced changes in DNA damage pathways in the VH.^39^

At the macro-level, the preventive effects of pre-trauma MPH on fear-related symptoms can be linked to the asymmetric effects of low-dose MPH within the cerebral hemispheres.^40, 41^ That is, while upregulation of left-hemisphere dopaminergic function is related to task accuracy and left-PFC control over working memory (WM) processes,^42^ single low doses of MPH were shown to normalize left-hemisphere dopaminergic underfunctioning in ADHD,^41^ and to upregulate functional activation in left-PFC regions involved in temporal processing^43^ and mediation of WM load.^40^ These lateralized effects become specifically relevant given that functional lateralization is strongly related to emotional regulation^44, 45^ and memory processing^8, 46^ in healthy human populations, and the intrusive nature of traumatic recollections^47, 48^ and severity of symptoms^49, 50^ in PTSD. Moreover, while in healthy subjects successful regulation of emotional responses during aversive learning involves left-hemisphere activity increment^51^ and selective left-hippocampus activation modulates the accuracy of its associative representations,^8^ cued-retrieval of traumatic representations and emotional flashbacks in PTSD were found to involve selective left-hemisphere underactivation^47, 48^ (see Hughes *et al.*^47^ for a comprehensive review). A concordant combination of the above can suggest that the lateralized effects of Pre-MPH might balance functional asymmetries involved in the processing of intense fear-emotions and their perceptual co-occurring space during the initial encoding and consolidation of an aversive experience. In consideration of the dual-role suggested for mixed lateralization, both as a risk-factor and the result of irrepressible re-experiencing of trauma-related fear in PTSD (see Ritov *et al.*^52^ for a comprehensive review), such lateralized-modulations, reducing tempospatial imprecisions in emotional representations, thus might subsequently prevent dysregulated fear-responses to contextual reminders of the aversive event in the long term.

In extreme situations, such as combat, the identification of environmental cues that signal genuine danger is critical. Under such circumstances, directing attention to threat cues may thus become highly adaptive.^53^ Generally applied immediately after trauma, in most prevention strategies for PTSD attention is typically trained away from threat cues under new safe circumstances.^54, 55^ However, differing from these common strategies, a recent trial by Wald *et al.*^53^ provide evidence that pre-trauma modification of attention to the direction of threat cues can actually reduce the risk for post-traumatic distress in the long-term. Utilizing attention bias modification training as a preventive strategy, this randomized controlled study trained soldiers to attend towards threat, in an attempt to enhance cognitive processing of potentially traumatic events. Indeed, when delivered prior to combat deployment, the pre-trauma attention bias modification training intervention mitigated the risk for PTSD following combat exposure.^53^ Revealing a pre-trauma window for PTSD prevention, the findings of this study may correspondingly point to the potential mechanisms involved in the preventive effects of pre-trauma MPH found here. In specifics, a possible key involvement of the attentional threat-monitoring system, and its optimal functionality, in the delicate balance between environmental demands and neurocognitive responses during trauma^53^ and the initial formation of its associative representations. When taken together, the detailed anatomical observations presented above illustrate a complex multidimensional circuitry, which may be involved in the preventive effects of pre-trauma MPH. Nevertheless, in line with the conceptual offer of Wald *et al.*,^53^ both micro- and macro-level observations indicate that pre-trauma MPH can selectively modulate basic attentional functions during trauma exposure. This modulation may preattentively bias emotio-cogntive processing towards cues that signal genuine danger. Furthermore, in such a case, the pre-trauma MPH could exert its preventive effects by promoting a more adaptive formation of associative representations of the traumatic experience. Given the temporally restricted impact of MPH and the low dose required for its preventive effects in this study, our findings may suggest a relatively simple low risk strategy for the prevention of PTSD. Particularly in populations such as combat and emergency response personnel that faces high risk for traumatic exposure within a timely defined framework. For example, during combat deployment, low-dose MPH can be selectively administered to soldiers headed for routine engagement in operational assignments.

To conclude, our behavioral data provide evidence that basic memory impairments play an important role in the development of fear-related pathology. This is in line with the hypothesized involvement of tempospatial–emotional inaccuracies in aversive representations in the pathogenesis of fear-related disorders. Bearing in mind the dose-dependent lateralized effects of MPH, and the significant connection between mixed lateralization and PTSD, our results may offer a new pharmacologic strategy of using low-dose Methylphenidate for the prevention of post-traumatic pathology in populations that are at high risk for traumatic exposures.
