# Supplementary figures and images for: Pre-trauma Methylphenidate in rats reduces PTSD-like reactions one month later
Source: Transl Psychiatry. 2017 Jan 10;7(1):e1000–. doi: 10.1038/tp.2016.277 (PMC5545737; doi:10.1038/tp.2016.277)

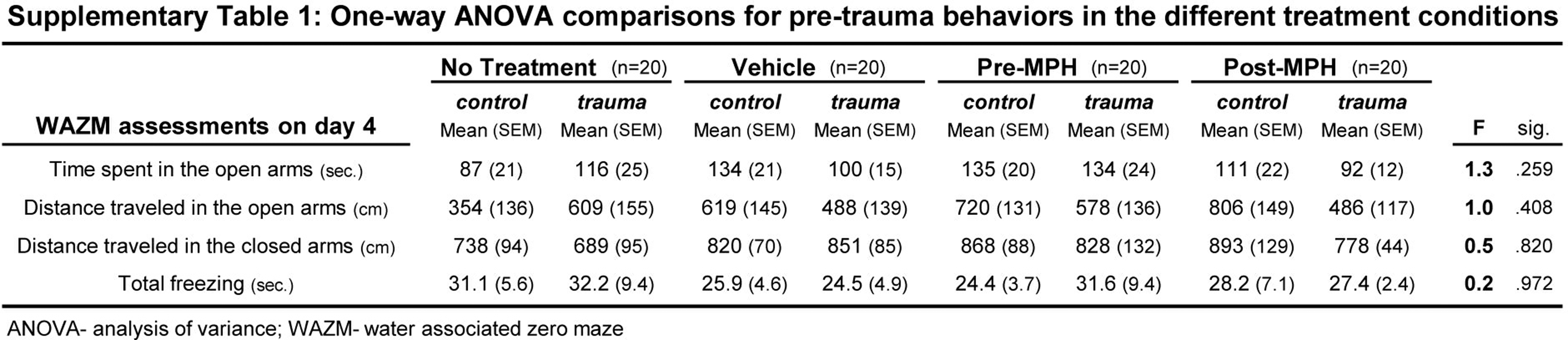

Supplement: Supplementary Table S1 [file tp2016277x1.tif]
